# Holiday Thoughts for Health Makers

**Published:** 1894-09-08

**Authors:** 


// The Hospital

An Institutional Journal of

The Medical Sciences and Hospital Administration, with

AN EXTRA NURSING SUPPLEMENT.

Vol. XVI.—No. 415.] [Sept. 8, 1894.

### Holiday Thoughts For Health Makers.

Health is the indispensable condition of all work of the best quality. The human brain is an instrument of so fine a temper that the smallest ill in any part of the body will take something from its working perfectness. The physician is the health maker. When he is separated for a period, as by an autumn holiday, from his daily work, he falls upon thinking, philosophising. He will ask himself such questions as these: What is health? Why are the different organs of the human body liable to those injurious changes of structure and function which we call disease? Why may a man not pass through the world without the risk of disease, and so be, at any rate, moderately happy? It is undoubted that disease plays a tremendous part in the world's affairs. It, rather than the fear of death in old age, is the real spectre which sits at all life's feasts. May we ever hope to annihilate disease? Never. May we ever hope to seriously diminish it, and to reduce it within controllable limits? If the general human brain greatly improves we may; if it remains stationary or goes backward we may not. An unintelligent world can never be a healthy world, however intelligent its physicians may be.

If disease plays so important a part in the world's affairs as we have affirmed, the class who treat disease, the medical class, must likewise occupy a significant place in the thoughts and practicalities of mankind. Medicine has done much for civilised man, but it has accomplished a mere fraction of what civilised man would like and expects it to do. Can it do any more? And in what direction should future effort point? It is obvious to the thinking medical man that medicine can do much more for men than she has hitherto done, or than she is even now doing. The most urgent call of the moment to the average medical practitioner is the command to master fully and thoroughly the knowledge which the pioneers of our profession have achieved, and to make the new knowledge, rather than the old hypotheses and traditions, the standard of everyday practice and work.

A plebiscite which would give us honestly and truly the exact ideals and methods of practice of the whole of the working profession of medicine would be profoundly interesting, and perhaps as startling as interesting. No worker does better work than his own ideals. It often strikes us that the ideals of the average doctor cannot be very high, since he neither enjoys unusually good health himself, nor does he live longer, on an average, than other classes of men who know nothing of scientific medicine. It is not likely that the average doctor has more real care for the well-being of his patient than he has for his own. If, therefore, his ideals are such as to permit him to be content with indifferent health and a briefer life than usual for himself, what must be the measure of really thorough and heartfelt care which he devotes to the well-being and longevity of his patients? One doubts the prophet who does not practice his own preaching, the physician who does not heal himself.

The period of cessation from daily strenuous work naturally disposes the thinking man to think, and the summer holiday gives leisure for considering any possible new departures for which the new autumn and winter may offer opportunities. The cultured doctor, of which we hope there are many thousands in the profession, is not a mere journeyman who works for weekly or annual wages. He is, or should be, an independent thinker, a man who can see and purpose, and carry his purpose into effect. The purposes of a man are governed by his ideals. Are there any great medical ideals at the present moment which are waiting to be realised? There are, as we have indicated, at least two which are urgent. The first is the improvement of the medical man's own health and brain power, the second the conscientious mastering of new knowledge and new principles, with a view to their immediate adoption in general practice. To these should be added the instruction of patients themselves in the general outlines and ideas of the new as distinguished from the old in medicine.

There is before the medical profession at the present moment a task of stern quality; no less a task than the conquest of the minds and fealties of all the intelligent portion of the community. The world is only partly with us in our attempts to master new knowledge and to compel success in practice. It ought to be entirely, enthusiastically with us. We must instruct and persuade it to our side. We cannot afford to do otherwise. Even from the point of view of our daily bread and butter we need the faith and sympathy of the non-professional world; we need it still more if the knowledge of health and disease is to make progress, and if methods of relief and cure are to be universally improved and practised. The average medical man is not enough of a thinker. He is too easily contented with making daily visits and taking daily fees. But the world asks him, nay, insists upon him at least trying, to think. If a routine practitioner were asked on a sudden what are the normal foundations of health, he would not be prepared with a rational answer. But an intelligent schoolboy of the modern period would tell us at once that inheritance, environment, and the right use of parts and organs are the normal foundations of health. Should the clever schoolboy be a better medical philosopher than even the least cultured of practising doctors?

An unarticulated skeleton, a higgledy-piggledy of unco ordinated specialisms—this is what the too eager and self-asserting young doctor of our period is bent upon converting medicine into. Not only is this the "note" of the young man in our own country and America, but even, though to a less extent, in philosophic Germany. It is, perhaps, inevitable that this should be so in such a time of lightning like change and discovery as our own. We are the sons of our professional fathers in the order of time; but in point of scientific knowledge our fathers were to us as the men of Mr. *Punch's* "Prehistoric Peeps" are to the Regent Street young man of the day. We forget that the latest developments of medical science are hardly a quarter of a century old. Our young men, and, indeed, many of our older men too, are crushed out of all intellectual form and proportion beneath the accumulating load of new knowledge, half knowledge, crude speculation, and daring therapeutic departures of the too instantly-present times. What is a quarter of a century in the lifetime of a long art and a profound science like that of medicine? What is it even in the life of the individual of weighty thought and deliberate life purpose? And yet our young specialists think they must make name and fame and fortune, not in twenty-five years, but in five; nay, some of them are still so near to their Eton jacket period in the quality of their thought that they imagine a single paper in a medical periodical is to make them famous for ever.

Against all this crudity, all this "raw haste," which, as Tennyson has told us, is "half-sister to delay," some strenuous protests have been uttered on both sides of the Atlantic. In America Dr. Jacobi has advised the budding specialist, who thinks by the study of a single organ of the body to raise himself above the shoulders of the ten times more learned physician, to bethink himself whether he is fit to practice medicine at all, even in one of the humblest of its departments. In England Sir Dyce Duckworth has planted his feet firmly on similar solid and rational ground. But the whole profession should "pull itself together," and ask itself what of enduring reasonableness there may be in many of the methods and professional-life strugglings of the present period? An autumn holiday, and a good one, is indispensable for the scientific doctor. If he would forget his own home labours and struggles during his brief period of rest, and let some such subjects as we have here briefly discussed simmer in his mind, he would be a wiser and a more clear-seeing man on his return to work, and the great profession to which he owes a whole-souled loyalty would be helped into clearer daylight and a worthier position among the nobler developments of our strenuous and upward-tending civilisation.

